# Polysubstance Abuse: Alcohol, Opioids and Benzodiazepines Require Coordinated Engagement by Society, Patients, and Physicians

**DOI:** 10.5811/westjem.2014.11.24720

**Published:** 2014-12-15

**Authors:** Uzor C. Ogbu, Shahram Lotfipour, Bharath Chakravarthy

**Affiliations:** University of California, Irvine, Department of Emergency Medicine, Orange, California

## Abstract

The Centers for Disease Control and Prevention (CDC) has published significant data trends related to substance abuse involving opioid pain relievers (OPR), benzodiazepines and alcohol in the United States. The CDC describes opioid misuse and abuse as an epidemic, with the use of OPR surpassing that of illicit drugs. Alcohol has also been a persistent problem and is associated with a number of emergency department visits and deaths independent of other substances. The use of these drugs in combination creates an additive effect with increased central nervous system suppression and a heightened risk of an overdose. We present a summary of the findings from the Morbidity and Mortality Weekly Report (MMWR) with commentary on strategies to combat prescription drug and alcohol abuse.

## CDC MMWR FINDINGS

In the October 10, 2014, issue of Morbidity and Mortality Weekly Report (MMWR), the Centers for Disease Control and Prevention (CDC) published data and trends related to emergency department (ED) visits and deaths associated with the combined use of alcohol, opioid pain relievers (OPR), and benzodiazepines.^1^ The MMWR article examined the overall trends, and age- and gender-specific trends in combined drug and alcohol use. They concluded that alcohol plays a significant role in ED visits and deaths associated with OPR and benzodiazepine misuse and abuse.

The CDC report used 2010 data from the Substance Abuse and Mental Health Services Administration’s Drug Abuse Warning Network (DAWN). DAWN monitors hospital ED visits (DAWN ED) and drug-related deaths (DAWN ME). The network collects data on illegal drugs, prescription and over-the-counter medications that contribute to an ED visit or death, and alcohol use associated with the event. A stratified random sample of DAWN ED data from 237 hospitals was used with hospital specific post-stratification weights applied. DAWN ME data were obtained from the 13 states (Delaware, Maine, Maryland, Massachusetts, New Hampshire, New Mexico, Oklahoma, Oregon, Rhode Island, Utah, Vermont, Virginia, and West Virginia) that provide data to DAWN.

In 2010, DAWN estimates indicate that of the 438,718 ED visits in the United States associated with OPR abuse, 18.5% (81,365) involved alcohol. Alcohol involvement was higher for ED visits related to benzodiazepine use with alcohol involvement noted in 27.2% (111,165) of the 408,021 ED visits. OPR-related ED visits involving alcohol were highest among persons aged 30 – 44 years (20.6%) and 45 – 54 years (20.0%). Benzodiazepine-related ED visits involving alcohol were highest among person aged 45 – 54 years (31.1%). ED visits with the combined use of alcohol, and OPR (81,365 visits) or benzodiazepines (111,165 visits) were more common among men (22.9%, 95% CI [18.7%–27.7%]) for OPR and 30.6%, 95% CI [26.7%–34.8%]) for benzodiazepines] than women 13.5%, 95% CI [11.1%–16.4%]) for OPR and 24.1%, 95% CI [19.6%–29.2%]) for benzodiazepines].

In 2010 of the 3,833 OPR-related deaths and 1,512 benzodiazepine-related deaths recorded in DAWN, 22.1% (860) and 26.1% (393) of deaths respectively involved alcohol. OPR-related deaths involving alcohol were highest among those aged 40–49 years (25.2%) and 50–59 years (25.3%). Benzodiazepine-related deaths were highest among those 60 years and older (27.7%).

The report highlights a few limitations of the study and the DAWN data, which include the completeness of the data, potential misclassification, and sampling limitations. Drug identification, amount of alcohol consumed, and subsequent inclusion in DAWN may be incomplete. The death data are limited to 13 states with varying triggers for coroner review, which is required for inclusion in DAWN ME. There is also variation in toxicology testing practices, which affects detection of drugs and inclusion in the DAWN ME database. In addition, DAWN data does not distinguish between medical and non-medical use.

## COMMENTARY

An estimated 181.7 million opioid prescriptions were written in 2012, representing a 33% increase from 2001.^2^ Sales of opioid medication in 2010 are estimated to be of sufficient volume to supply every adult American with 5mg of hydrocodone every six hours for 45 days.^3^ Benzodiazepines have long been among the most prescribed psychoactive agents.^4^ The combination of both drugs has become a cause for growing concern with a recent study indicating that among chronic pain patients a significant proportion tested positive for benzodiazepine metabolites in their urine.^4^ The lifetime prevalence of alcohol abuse is 17.8%, with a large overlap with the abuse of other substances.^5^ The combined use of OPR, benzodiazepine, and alcohol potentiates the effect sought by the user but also exposes them to significant additional risk of adverse events, even when the individual agents are used as prescribed.^6^,^7^ The ED is the frontline for the treatment of these adverse events despite ED physicians prescribing fewer pills and less potent opioid formulations than office-based physicians.^8^

The commercial availability of alcohol requires that efforts to combat alcohol abuse focus on the individual. Public service announcements advocate drinking responsibly, legal statutes prescribe a maximum allowed blood-alcohol concentration, and screening and brief interventions identify individuals at risk and attempt to get them into treatment. OPR and benzodiazepines are controlled substances that require a prescription to obtain. Efforts to combat abuse of these substances have largely focused on limiting their availability, identification of those at risk, and referral for treatment. Prescription drug monitoring programs (PDMP) were established to combat doctor shopping and identify potentially dangerous interactions such as concurrent prescriptions for an OPR and benzodiazepines. However, technical issues such as lack of interstate interoperability -and the limited penetration among prescribing physicians have limited the effectiveness of state-based PDMP.^9^ OPR prescribing guidelines have become a new tool in some states such as New York and Washington where there are limits on the number of pills a physician is allowed to prescribe.^10^,^11^ However, anecdotal evidence suggests that these efforts, while effective, result in a change in abuse behavior.^12^,^13^ Drug-seeking patients cross state lines to obtain drugs, switch to illicit drugs, or find non-physician sources for opioids. A recent report indicates that heroin use and heroin-related adverse events have increased in states such as California, Florida, Kentucky, Massachusetts, New York, Ohio, Oregon, and Washington alongside a decline in the availability of OPR.^14^

At the center of this polysubstance misuse and abuse problem is an individual. Individuals involved in adverse events associated with OPR, benzodiazepine, and alcohol can be placed along a spectrum. On one end are short-term users with acute pain who were prescribed these drugs and attempt to use them as intended but are unaware of the full scope of potential adverse events. At the other extreme are those who are aware of the risks and willfully neglect these risks in the throes of their dependence or addiction. Among other groups between these extremes are non-patients, such as children and other family members, who improperly obtain these substances to experiment with them. Abuse prevention efforts need to be addressed on multiple levels that include the society, patients, and physicians. While a number of organizations have conducted community-based interventions aimed at raising awareness of prescription drug abuse, these initiatives are often interpreted as being targeted at ‘abusers’ and concerned parents. The physician-encounter that leads to an OPR prescription for acute pain represents an important teachable moment when patients and their families can be educated about their medications, side effects and potential interactions. However, lack of time limits a physician’s ability to capitalize on these moments, especially in the ED. For example, studies of discharge instructions indicate that few patients (<20%) are aware of what to do with unused medication.^15^,^16^ Sharing of leftover opioid pills is common among patients who often retain them for later use.^17^,^18^ This contributes to estimated diversion rates as high as 29% in young adults and college students.^19^

Prescribed OPR pills may flow through a number of consumption pathways from appropriate use to misuse and diversion. Adequate patient education about their medications represents an additional opportunity to disrupt the pipeline of early-stage non-medical use of opioids, complementing policies aimed solely at physicians. Among short-term users, the risk of dependency and addiction is small with appropriate use. However, in a recent analysis of the source of opoids among those reporting non-medical use in the past year, 70% reported obtaining drugs (free, stolen, or bought) from friends or family members, approximately 20% from a physician and 10% from other illicit sources.^20^ Among those reporting use lasting only 1–29 days the portion identifying a friend or family member as a source was approximately 75%.

The expansion of access to health insurance increases the number of at-risk individuals by increasing access to prescription medications. However, it also represents an opportunity to educate more patients and families. A White House policy document pinpoints education, tracking and monitoring, proper medication disposal, and enforcement as key approaches to combating the opioid epidemic.^21^ However, education interventions have focused on physicians and pharmacists. Few interventions aim to change the attitudes and behavior of patients towards opioids.^22^,^23^ This information gap is exacerbated by direct marketing campaigns that highlight the potential benefits of opioids.^24^ Patients assume that medications prescribed by their doctor are safe and pose little risk. Detailed discussion of the potential for addiction, the need for safe storage and disposal, and an in-depth exploration of alternative treatments is often neglected, particularly for non-chronic pain patients. A recent study attributes medication-related adverse events to host factors and environmental factors such as patient health literacy, awareness of how to use medications, awareness of side effects, and multiple prescribers and limited communication among prescribing physicians.^25^ The communication failure may be due to the manner in which the information is presented. Warning labels, medication sheets, and prescribing information are presented on packaging, pill bottles, or with discharge instructions. These text-dense sources are not read, ignored, or not fully comprehended by patients.^26^–^28^ Future research should develop effective tools for communicating medication information to a wide audience of patients acknowledging differences in health literacy and levels of engagement. Alternative forms of communication such as short multimedia videos, text messages or social media should be explored. Such efforts will add an additional front to the battle against substance abuse and reduce the number of unintentional injuries, deaths, and ED visits associated with prescription drug abuse.

## References

[1] Jones CM, Paulozzi LJ, Mack KA (2014). Alcohol involvement in opioid pain reliever and benzodiazepine drug abuse-related emergency department visits and drug-related deaths - United States, 2010. MMWR Morb Mortal Wkly Rep.

[2] New York Times (2013). The Soaring Cost of the Opioid Economy.

[3] Manchikanti L, Helm S, Fellows B (2012). Opioid epidemic in the United States. Pain Physician.

[4] Jones JD, Mogali S, Comer SD (2012). Polydrug abuse: a review of opioid and benzodiazepine combination use. Drug Alcohol Depend.

[5] Merikangas KR, McClair VL (2012). Epidemiology of substance use disorders. Human genetics.

[6] Beauchamp GA, Winstanley EL, Ryan SA (2014). Moving beyond misuse and diversion: the urgent need to consider the role of iatrogenic addiction in the current opioid epidemic. Am J Public Health.

[7] French DD, Spehar AM, Campbell RR, Henriksen K, Battles JB, Marks ES (2005). Outpatient Benzodiazepine Prescribing, Adverse Events, and Costs. Advances in Patient Safety: From Research to Implementation (Volume 1: Research Findings).

[8] Menchine M, Axeen S, Plantmason L (2014). Strength and dose of opioids prescribed from US emergency departments compared po office practices: implications for emergency department safe-prescribing guidelines. Ann Emerg Med.

[9] Clark T, Eadie J, Kreiner P (2012). Prescription drug monitoring programs: an assessment of the evidence for best practices. Report.

[10] Chu J, Farmer B, Ginsburg B, New York City Emergency Department Discharge Opioid Prescribing Guidelines Clinical Advisory Group (2013). New York City Emergency Department Discharge Opioid Prescribing Guidelines.

[11] Neven DE, Sabel JC, Howell DN (2012). The development of the Washington State emergency department opioid prescribing guidelines. J Med Toxicol.

[12] Unick GJ, Rosenblum D, Mars S (2013). Intertwined epidemics: national demographic trends in hospitalizations for heroin- and opioid-related overdoses, 1993–2009. PLoS One.

[13] Mars SG, Bourgois P, Karandinos G (2014). “Every ‘never’ I ever said came true”: transitions from opioid pills to heroin injecting. Int J Drug Policy.

[14] Huecker MR, Shoff HW (2014). The law of unintended consequences: illicit for licit narcotic substitution. West J Emerg Med.

[15] Bates C, Laciak R, Southwick A (2011). Overprescription of postoperative narcotics: a look at postoperative pain medication delivery, consumption and disposal in urological practice. J Urol.

[16] Wieczorkiewicz SM, Kassamali Z, Danziger LH (2013). Behind closed doors: medication storage and disposal in the home. Ann Pharmacother.

[17] Lewis ET, Cucciare MA, Trafton JA (2013;2014). What do patients do with unused opioid medications?. Clin J Pain.

[18] Wallace LS, Wexler RK, Miser WF (2013). Development and validation of the patient opioid education measure. J Pain Res.

[19] Arria AM, Garnier-Dykstra LM, Caldeira KM (2011). Prescription analgesic use among young adults: adherence to physician instructions and diversion. Pain Med.

[20] Jones CM, Paulozzi LJ, Mack KA (2014). Sources of prescription opioid pain relievers by frequency of past-year nonmedical use United States, 2008–2011. JAMA Intern Med.

[21] Office of National Drug Control Policy (2011). Epidemic: responding to America’s prescription drug abuse crisis.

[22] McCauley JL, Back SE, Brady KT (2013). Pilot of a brief, web-based educational intervention targeting safe storage and disposal of prescription opioids. Addict Behav.

[23] Volkow ND, McLellan TA (2011). Curtailing diversion and abuse of opioid analgesics without jeopardizing pain treatment. JAMA.

[24] Van Zee A (2009). The promotion and marketing of oxycontin: commercial triumph, public health tragedy. Am J Public Health.

[25] Gertler SA, Coralic Z, Lopez A (2014). Root Cause Analysis of Ambulatory Adverse Drug Events That Present to the Emergency Department. J Patient Saf.

[26] Gill PS (2013). Prescription painkillers and controlled substances: an appraisal of drug information provided by six US pharmacies. Drug Healthc Patient Saf.

[27] Wolf MS, King J, Wilson EA (2012). Usability of FDA-approved medication guides. J Gen Intern Med.

[28] Wallace LS, Keenum AJ, Roskos SE (2008). Suitability and readability of consumer medical information accompanying prescription medication samples. Patient Educ Couns.

